# An overview of caspase: Apoptotic protein for silicosis

**DOI:** 10.4103/0019-5278.72237

**Published:** 2010-08

**Authors:** Rajani G. Tumane, Shubhangi K. Pingle, Aruna A. Jawade, Nirmalendu N. Nath

**Affiliations:** Department of Biochemistry, National Institute of Miners’ Health, JNARDDC Campus Wadi, Nagpur, India; 1 Department of Biochemistry, L. I. T, R.T.M. University, Nagpur, India

**Keywords:** Apoptosis, caspase, DNA adduction, Fas/FasL, occupational diseases and silicosis

## Abstract

Silicosis is a chronic lung disease characterized by granulomatous and fibrotic lesions, which occurs due to accumulation of respirable silica mineral particles. Apoptosis is an important phenomenon of cell death in silicosis. The relationship between silica dust and its exposure is well established. But, the complex chain of cellular responses, which leads to caspase activation in silicosis, has not been fully discovered. Caspase activation plays a central role in the execution of apoptosis. Silica-induced apoptosis of the alveolar macrophages could potentially favor a proinflammatory state, occurring in the lungs of silicotic patients, resulting in the activation of caspase prior to induction of the intrinsic and extrinsic apoptosis pathways. Recent studies indicated that apoptosis may involve in pulmonary disorders. This review summarizes the current knowledge about the underling mechanism of biochemical pathways in caspase activation that have been ignored so far in silicosis. In addition, caspase could be a key apoptotic protein that can be used as an effective biomarker for the study of occupational diseases. It may provide an important link in understanding the molecular mechanisms of silica-induced lung pathogenesis.

## INTRODUCTION

In India, there are three million people exposed to silica in mines and industries. Scientists have shown a high prevalence of silicosis in small factories like stone cutting, slate pencil, foundry work, rock grinding and tunneling etc. More than 1,00,000 workers encountered high-risk silica exposure through mining operations. Silica is found to be a potent occupational fibrogenic agent that is capable of inducing apoptosis in alveolar macrophages (AMs).[1] After its exposure, AM gets activated, which enhance oxidative stress in the cells. Deposited silica particles in the lungs damage protein, lipid, macrophages, epithelial cells and DNA. It results in the release of a wide variety of enzymes and inflammatory cytokines. These inflammatory cytokines induce the late fibrogenic reaction in coal workers. These consequences may up-regulate the antioxidant enzymes and release reactive oxygen species (ROS) and nitrogen species (RNS).[2] These factors are responsible for different occupational diseases such as adult respiratory distress syndrome (ARDS), emphysema, chronic obstructive pulmonary disease (COPD), chronic bronchitis and lung fibrosis.[3]

Caspases are cysteinyl aspartate-specific proteases, which belongs to the family C14. Its activation occurs through proteolytic cleavage at the conserved aspartic acid residues. Catalytically active caspases exhibit narrow substrate specificity for cleaving their substrates after aspartic acid residues. The first caspase was identified as a protease responsible for activating the precursor of interleukin-1b-converting enzyme (ICE). Caspases 2, 3, 6, 7, 8, 9 and 10 are involved in the regulation and execution of apoptosis. Caspases 1, 4 and 5 are involved in the activation of inflammatory cytokine. Caspases 2, 8 and 10 are apoptotic initiator caspases while caspases 3, 6 and 7 are cell death executioners.[4–8]

Silica particles induce apoptosis in the lung, but it requires specific molecular mechanisms. It is typically mediated through a hierarchy of caspase activation. The activated caspase is governed by a coordinated hierarchy of initiator caspases (e.g., caspases 8 and 9) that activate effector caspase (e.g., caspase 3).[9] These are responsible for apoptosis via proteolytic cleavage of homeostatic and structural proteins. Some protein from the tumor necrosis factor family such as myc and Fas (also called APO-1 or CD95) can trigger apoptosis. Apoptosis is mediated by Fas/Fas ligand (FasL) interaction, which is important to maintain the immune system against tolerance to antigens in silicosis.[10] Thus, it is important to understand how silica induces caspase activation in silicosis, leading to subsequent apoptosis, which still remains unclear. It is better to understand the cellular pathway that is responsible for caspase activation. It is needed to explore apoptotic factors that are responsible for caspase activation in silicosis and potential factors that may be used as biomarkers for early diagnosis of silicosis. However, the use of biomarker can change, and greatly enhance, the process of risk assessment. Recently, considerable attention has been given by the scientific community to the utilization of biomarkers in the early diagnosis and prognosis of occupational diseases, which prevents further health deterioration in silicotic individuals. This review summarizes the role of different caspases in silica-induced apoptosis.

## CASPASE ACTIVATION IN SILICA-INDUCED APOPTOSIS

It has been well documented that AMs get activated when exposed to silica particles in the lungs, which results in the release of macrophage products like fibrogenic factors, lysosomal enzymes, free radicals and cytokines. The study indicated that silica is able to induce apoptosis in AMs, while relatively little is known about the underlying mechanisms involved, based on the fact that silica is able to induce ROS formation, followed by caspase-9 and caspase-3 activation, poly ADP-ribose polymerase (PARP) cleavage, DNA fragmentation and oxidative stress in AMs. Oxidative stress is an important mediator of apoptosis and, therefore, investigators have more interest to investigate the role of ROS and oxidative stress in silica-induced apoptosis *in vivo* and *in vitro* in AMs.[11–14] The silica exposure may acts as an initiator of caspase activation by the mitochondrial pathway and release cytochrome c. Once Cytochrome c is released from the mitochondria. It works together with other cytosolic protein factors, Apaf-1 (apoptosis protease activating factor) and caspases-9, which activates caspase-3 and PARP cleavage to execute the apoptotic process. This consequence is responsible for caspase activation and apoptotic cell death. The caspase-3-, caspase-9- and Apaf1-knockout mice showed a similar phenotype, with abnormal brain development. These findings lend further support to the hypothesis that caspase-3 plays an effector role in neuronal cell death during normal development of the brain. Thus, attention has been focused on the role of caspase-3 in neurodegenerative disorders such as stroke, Alzheimer disease and Huntington disease. Silica-induced caspase-9 activation in rat AMs, as measured by 7-amino-4-trifluoromethyl coumarin (AFC) fluorescence intensity, liberated from its substrate N-acetyl-Leu-Glu-His-Asp-AFC (Ac-LEHD-AFC). Silica-induced caspase-3 activation in rat AMs, as measured by 7-amino-4-methylcoumarin (AMC) fluorescence intensity, cleaved from its substrate N-acetyl-Asp-Glu-Val-Asp-AMC (Ac-DEVD-AMC). In a dose-response study, elevated AFC fluorescence intensity was detected even with the lowest concentration of silica. In a dose-response study, significant increase in AMC fluorescence intensity resulted from the cleavage of caspase-3 substrate N-acetyl-Asp-Glu-Val-Asp-AMC (Ac- DEVD-AMC).[15–19] Silica-induced apoptosis is attenuated by N-acetyl-Asp-Glu-Val-Asp aldehyde and ebselen as a potent antioxidant and also as a caspase-3 inhibitor.[20,21] It might be expected that silica-induced apoptosis occurs during resolution of the inflammatory response. Extensive apoptosis was observed in silica-induced inflammatory infiltrates in the lung parenchyma of silica-exposed mice. Leigh *et al*. stated that silica-induced apoptosis in the lung tissues has a regulatory role in the process of inflammation by maintaining stable levels of neutrophils in the inflammatory sites and attracting more AMs into the alveolar space to engulf apoptotic cells.[22] Scientists have implicated that the caspase inhibitor reduces intrapulmonary neutrophil accumulation and lung inflammation in their *in vivo* experiment. It suggested that activated caspases have a specific role in the inflammatory process.[23,24] But, the mechanisms involved in the silica-induced apoptosis in the carcinogenesis and pathogenesis are unclear. Uncontrolled regulations of apoptosis have been observed in a variety of human diseases, including cancer, autoimmune diseases and neurodegenerative disorders. On exposure of monkeys (*Macacus cynomolgus*) to quartz aerosols for 18 weeks, X-ray changes corresponding to the presence of cellular silicotic nodules were observed between 21 and 64 weeks after the end of the dust exposure. Compared with untreated monkeys, various modifications were reported, such as increased release of superoxide anion and increased levels of a1-PI and lysosomal glycosidases, while free elastase-like activity was detected in BAL fluids.[25,26]

It was reported that silica particles, but not quartz anatase titanium dioxide, activated caspase-3 in rat macrophages and caspases 1, 3, 6 and 9 in mouse AMs cell line (MH-S cells).[27,28] The *in vitro* model of silica-induced apoptosis shows that silica particles affect lysosomal membrane integrity by activation of intracellular pathways in mouse AMs, leading to caspase activation and apoptosis.[29] The mitochondrial membrane permeability can be measured by the release of cytochrome c or depolarization of the inner mitochondrial membrane. Cyclosporin A, an inhibitor of mitochondrial permeability, decreased, partially, the mitochondrial depolarization as well as activation of caspases-3 and -9, indicating the possible mitochondrial dysfunction after silica exposure *in vitro*.[30,31] Increased expression of the antiapoptotic protein Bcl-xL, which is found in caspase cascade, suggested that macrophages may show prolonged survival in patients with chronic lung diseases.[32] Although significant gaps in knowledge about caspases remain, cellular pathways leading to silica-induced caspase activation have partly been defined. Numerous studies have been performed by scientists related to ROS, RNS, tumor necrosis factor (TNF), nuclear transcription factor-kβ (NF-kβ) and oxidative stress in apoptosis, but fewer studies was carried out for caspases in silicosis. These findings suggest that apoptosis is an important form of cell death caused by silica exposure, in which the elevated ROS level may act as an initiator, leading to caspase activation and PARP cleavage to execute the apoptotic process.

## FAS, FASL AND CASPASE-MEDIATED APOPTOSIS IN SILICOSIS

Fas (CD95) is a membrane bound and shed protein that belongs to the TNF gene family. It is constitutively expressed by lung epithelial cells, while FasL is highly expressed on CD4, CD56 and CD45RO broncheoalveolar lavage cells. FasL expression is tightly regulated and induced by NF-kβ.[33] Tumor-expressing FasL was destroyed by infiltrating granulocytes observed *in vivo*, considered being involved in the pathogenesis of autoimmune disease.[34,35] In silicosis patients, apoptosis is initiated when Fas is engaged by Fas trimers. The Fas-associated death domain (FADD), adaptor protein, associates with multimerized Fas and recruits procaspase 8 to form the death-inducing signaling complex (DISC). The procaspase 8 activate each other and induce the caspase cascade and apoptosis pathway.[36] It is well established that caspase-8 may have an additional role in the immune system, promoting lymphocyte activation and T cell proliferation. Thereafter, activated caspase-8 triggers a caspase-cascade involving the activation of CAD/CPAN/DFF40 (by removing its inhibitor, ICAD/DFF45), DNA fragmentation and, finally, apoptotic cell death.[37,38]

Lymphocyte apoptosis mediated by Fas/FasL interaction regulates immune response and FasL-mediated apoptosis of leukocytes prevents inflammatory reactions at immune-privileged sites. Thus, FasL exerts a proinflammatory role.[39–41] Increased apoptosis of inflammatory cells has been shown to colocalize with increased expression of FasL in silicotic lungs. Apoptosis in the silicosis can be blocked by the neutralizing anti-FasL antibody *in vivo*.[42,43]

Silica-induced pulmonary inflammation requires FasL expression; FasL-expressing pulmonary macrophages attract neutrophils and initiate silicosis. Scientists observed that silica particles exposed to wild-type mice develop pulmonary inflammation, with production of TNF-α and interstitial neutrophils and macrophage infiltration in the lung. Strikingly, FasL-deficient generalized lymphoproliferative disease mutant (Gld) mice did not develop silicosis. Gld mice had markedly reduced neutrophil extravasations into the broncheoalveolar space and did not show increased TNF-α production, nor did they develop silicosis due to lacked pulmonary inflammation. Silica-induced Fas ligand expressions in lung macrophages *in vitro* and *in vivo* promote FasL-dependent macrophages apoptosis. Thus, FasL plays a central role in the induction of pulmonary silicosis.[44]

To understand the role of apoptosis in silicosis through the Fas/FasL interaction, scientists had taken samples from the broncheoalveolar lavage fluid and found the expression of Fas antigen, FasL by the apoptosis of cytotoxic effector and memory cells. In the pathogenesis of silicosis, the Fas/FasL system is implicated in the inflammatory process.[45] Treatment with the procaspase inhibitor Z-VAD-FMK (Z, benzyloxycarbonyl; FMK, fluoromethylketone) significantly decreased lung fibrosis. Besides triggering apoptosis via caspase-8 activation, Fas engagement also induces secretion of IL-1b and IL-8, resulting in neutrophil extravasations, and supporting a major chemotactic role for FasL. It is possible that the generated substances counteract with the increase of cell number and regulate the silica-induced effects through the induction of apoptosis.[36] It was demonstrated that high level of serum-soluble Fas (sFas) is found in patients with silicosis, systemic sclerosis (SSC) and systemic lupus erythematosus (SLE) without any clinical symptoms of autoimmune disease in peripheral blood mononuclear cells (PBMC).[46,47] Reduced expression of inhibitory genes for Fas-mediated apoptosis in PBMC derived from silicosis patients. These results and previous investigations indicated the existence of a lymphocyte fraction that is resistant to Fas-mediated apoptosis, suggesting the presence of two fractions of lymphocytes in silicosis patients. To prevent not only respiratory diseases but also immunological disorders in silicosis patients, further study into the role of Fas-mediated apoptosis in these patients is required.[48] It seems that imbalance between apoptotic and proapoptotic factors could be responsible not only for immunologic disturbances and inflammation but also for internal organ involvement in the course of systematic sclerosis as well, just like in SLE.

**Figure 1:**
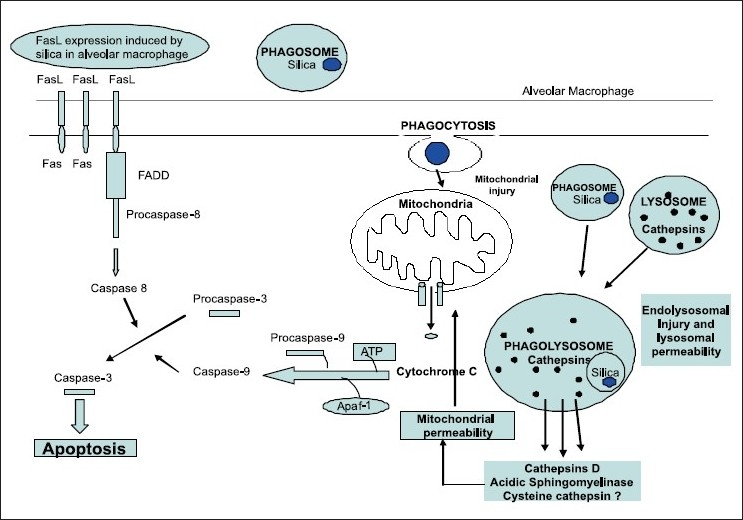
The extrinsic and intrinsic pathways of silica-induced apoptosis Fas, death receptor; Fas ligand (Fas L), Fas-associating protein with death domain; (FADD), apoptotic protease-activating factor-1 (Apaf-1)

A new member of the human CARD-containing family of proteins, it has a high degree of homology to the CARD domain of caspase-1 and can bind to caspase-1 and its related proteins, pseudo-ICE and ICEBERG. CARD-8 attenuates ICE activity, thereby decreasing IL-1β secretion. A reverse transcriptase-polymerase chain reaction study revealed that CARD-8 has the same pattern of expression as caspase-1. CARD-8 can also negatively regulate NF-κB activation by diverse stimuli, suggesting that this protein may control cell survival. Consistent with these results, stable expression of CARD-8 sensitizes cells to differentiation-induced apoptosis. Furthermore, overexpression of CARD-8 can induce apoptosis in transfected cells. Although the precise function of CARD-8 is not clear, it was suggested that it may function as an adaptor molecule regulating caspase-1 activation (IL-1 β production), NF-κB activation and apoptosis.[49] Therefore, Fas/FasL and caspase-mediated apoptosis in silicosis not only provide clues for the pathogenesis and treatment of immunological disorders but also aid in predicting the preclinical status of complicated autoimmune diseases found in occupational disease. Summary of the currently delineated cellular pathways leading to silica-induced apoptosis has been shown in Figure 1.

## CASPASE ACTIVATION AND DNA ADDUCTION IN SILICOSIS

The mechanisms of apoptosis involved in DNA adduction in silicosis have not been fully established. A group of cysteine proteases have been identified in both *Caenorhabditis elegans* (ced-3) and mammalian cells (caspases) as playing a critical role in the apoptotic process.[50–52] Among the various caspases, caspase-9 and caspase-3 appear to be involved in DNA adduction.[53,54] Procaspase-9 is an essential part of the “apoptosome,” resulting in activation of caspase-9.[55,56] The activated caspase-9 cleaves caspase-3 to orchestrate the biochemical execution of apoptosis. Caspase-3 is one of the major effector caspases and plays a critical role in the characteristic apoptotic changes, including chromatin condensation, DNA fragmentation and formation of apoptotic bodies.[54] It is observed that bcl-2 and Apaf-1 play an important role in the formation of apoptosome, leading to the activation of the caspase pathway.[57] Recently, it was reported that low levels of Apaf-1 protein can determine sensitivity to apoptosis downstream of mitochondrial events, suggesting that regulation of Apaf-1 may be important for apoptosis regulation. However, it seems that caspase activation and subsequent apoptosis could still occur in an Apaf-1-dependent pathway.[58] In addition, DNA fragmentation during apoptosis is mediated by a heterodimeric protein complex composed of DFF45/ICAD24) and DFF40/CAD/CPAN25).[59,60] These protein complexes are located downstream of caspase-3 to trigger DNA fragmentation and change the chromatin structure. But, it has been reported that DFF45 functions as an inhibitory factor in caspase-sensitive nuclease. In any case, DFF45 and CPAN possess regulatory activity for DNA fragmentation induced by caspase activation.[61,62] These indicate that caspases are involved in the apoptosis in different occupational diseases like pulmonary disorders, acute lung injury, diffused alveolar damage, idiopathic pulmonary fibrosis and other lung disorders caused by bleomycin, silica, endotoxin and the deposition of immune complexes.

## DIFFERENT TECHNIQUES AND SPECIMENS TO DETECT CASPASES

### Electron Microscopy

- Immunoelectron microscopy revealed immunogold labeling for caspases 3 and 8 in the mitochondria with the accumulation of caspase-3 in the apoptotic bodies.
- Examination of the cells with electron microscopy confirmed that the active caspase-3-containing nuclei in the proliferative regions often had infoldings and appeared to be undergoing division. Some of the cells with active caspase-3-labeled nuclei in the bulb had synapses on their somata or dendrites. Labeled dendritic spines and a few axon terminals were also observed in the olfactory bulb.

### Enzyme-Linked Immunosorbent Assay (ELISA)

- Thymus, spleen and lymph nodes were removed for the determination of caspase-3 expression by ELISA.
- We describe an ELISA for quantifying relative amounts of active caspase-3 in apoptotic cells.

### Flow Cytometry

- Expressions of survivin and caspase-3 in 101 cases of esophageal squamous cell carcinoma (ESCC) were quantitatively detected with flow cytometry.
- A highly sensitive flow cytometry-based cytotoxic T-lymphocyte (CTL) assay using the cleavage of caspase-3 in target cells as a readout.
- Flow cytometry detection of caspase-3 activation in preapoptotic leukemic cells.

### Immunocytochemistry

- Immunohistochemistry was performed for the detection of expression of c-Myc and caspase-3 in the A375 cells.
- Expression of survivin and caspase-3 was evaluated using immunocytochemistry on oral cancer cells induced by staurosporine.

### Immunofluorescence

- Fluorescence-labeled inhibitor of caspases (FLICA) was used to detect caspase-3 activity in apoptotic cells in this project; cell morphology and caspase-3 sublocalization were determined by confocal microscopy.
- Apoptosis was assessed by immunofluorescent detection of the active form of caspase-3. this assay being validated with peripheral blood neutrophils.

### Immunohistochemistry

- Terminal deoxynucleotidyl transferease-mediated dUTP-biotin nick end labeling (TUNEL) technique as well as immunohistochemical studies were used to evaluate the expression of caspase-3 and caspase-6 of breast cancer and the proliferation index.
- Apoptosis in various forms of lupus nephritis LN and its relationship to histomorphological changes and selected laboratory findings was studied using activated caspase-3 as a novel marker of apoptosis. Immunohistochemical analysis showed a marked induction of caspase-3 (p20) in every injured group compared with normal controls.
- To assess the underlying age-related changes in the cellular distribution of caspase-3, Scientist examined the motor cortex, cerebellum and hippocampus of young (4-year-old, *n* = 4) and old (20-year-old, *n* = 4) rhesus monkeys by immunohistochemistry.
- Expression of caspase-3, Bcl-2 and p53 was evaluated by immunohistochemistry in generalized aggressive periodontitis.
- Pattern of caspase-3 expression in noncancerous, premalignant (atrophic gastritis and intestinal metaplasia) tissue and cancer spots were analyzed under the same experimental conditions using immunohistochemistry.
- To detect apoptosis of germ cells and expression of caspase-3, TUNEL assay and immunohistochemistry (SABC) were used, respectively.
- Immunohistochemical expression was tested in 40 cases of BE, including 11 low-grade and 19 high-grade dysplasias (HGD), and samples were obtained from 40 surgical specimens of esophagectomy performed for HGD or Barrett’s adenocarcinoma.
- Immunohistochemistry was used to detect the protein expression of COX-2 and caspase-3 in the bronchial endothelial cells.
- Immunohistochemistry was performed by a sensitive peroxidase–streptavidin method on formalin-fixed, paraffin-embedded myocardial infarction tissue using monoclonal antibodies against activated (cleaved) caspase-3.
- Semiquantitative immunohistochemistry revealed cleaved caspase-3-positive cells in human temporal lobe epilepsy (TLE) sections but not in controls.
- The cleaved caspase-3 immunohistochemistry detected apoptosis of the lymphoma cells most sensitively compared with TUNEL and ssDNA immunohistochemistry.
- Apoptotic rates were measured using hematoxylin and eosin morphological assessment and immunohistochemical staining with antibodies to activated caspase-3 and M30.
- Immunohistochemistry for activated caspase-3 and TUNEL was performed on the trigeminal ganglion after infraorbital nerve transection in newborn rats.
- Caspase-3 expression in human gastric carcinoma to clarify the clinicopathological importance and investigation of the apoptosis avoidance mechanism by an immunohistological method. Actual activation of caspase-3 was determined by immunohistochemistry using monoclonal antibody that recognizes only activated caspase-3.
- Apoptosis in normal lymphoid organs from healthy, normal, conventional pigs at different ages detected by TUNEL and cleaved caspase-3 immunohistochemistry in paraffin-embedded tissues.
- Formalin-fixed paraffin-embedded tissues from 56 cases of primary gastric malignant lymphoma and their adjacent nontumor mucosa were evaluated for PTEN and caspase-3 protein expression by streptavidin–biotin complex (SABC) immunohistochemistry.
- Immunohistochemistry (SP method) was used to determine the expression of caspase-3 and Bcl-2 in 52 cases of BTCC and 10 normal bladder mucosas.
- The intra-islet expression of caspase-3 in the NOD mouse was examined immunohistochemically following acceleration of the disease with cyclophosphamide. Caspase-3 expression was studied in adjacent epithelial cells, cancer cells and lymphocytes of primary foci and cancer cells of metastatic foci from 113 cases of gastric cancer by streptavidin–biotin–peroxidase (S-P) immunohistochemistry.
- Comparison of immunohistochemistry for activated caspase-3 and cleaved cytokeratin 18 with the TUNEL method for quantification of apoptosis in histological sections of PC-3 subcutaneous xenografts.
- Immunohistochemical localization of active caspase-3 in the mouse ovary: growth and atresia of small follicles.
- Immunohistochemical and biochemical assessment of caspase-3 activation and DNA fragmentation following transient focal ischemia in the rat.
- Caspase-3 expression was examined immunohistochemically using a polyclonal antibody that recognized uncleaved caspase-3 in pathologic stage I, nonsmall cell lung cancer.
- Measurement of apoptosis by immunohistochemistry using an antibody against the active form of caspase 3 is therefore reliable and correlates strongly with morphological assessment.
- Immunohistochemical analysis was performed on formalin-fixed paraffin-embedded sections to assess caspase-3 expression.

### Western Blot

- Level of caspase-3 cleaved fragments was analyzed by Western blotting.
- Samples of the cerebral cortex, cerebellum, spinal cord and sciatic nerves were collected and examined for bcl-2, bax and caspase-3 expression using Western blotting.
- Liver MMP-9, TGF-beta1 and caspase-3 levels were quantified by Western immunoblotting.
- Western blot analysis was performed using antibodies against the pro- and active forms of caspase-3 and -9 or PARP in apoptosis of human granulosa-luteal cells.
- Caspase-3 in microparticles (EMP) from endothelial cells was studied using Western blot (*n* = 6) and flow cytometry (*n* = 6).
- Caspase-3 activity were determined by Western blot in 15 controls and 10 patients with benign prostatic hyperplasia and reported in a blinded fashion.
- To determine activation of the apoptotic regulatory cell proteins, caspase-3 and cleavage of PARP into its 85-kDa fragments were assessed by Western blotting.
- Western blot analysis was used to characterize the expression of caspase-3, caspase-8, PARP and p53 proteins in IM from 35 patients.
- Caspase-3 activation and PARP cleavage in lymphocytes exposed to Cr (III) complexes is revealed through Western blotting analysis.
- Active caspase-3 in the mucosa was detected according to the methods of immunohistochemistry and Western blotting.
- Immunohistochemistry and Western blot analyses were used to analyze the expression of caspase-3 in 40 archival specimens of patients with primary respected ESCC.

## CONCLUSION

In this review, we have summarized different biochemical pathways in which silica particle exposure can initiate apoptotic cell death of macrophages by the activation of caspases. Apoptosis plays a central role in silicosis by activation of different caspase-mediated pathways, such as mitochondrial and cell surface death receptor (Fas/FasL) pathway. In future, the challenge ahead is to map the functions of newly found apoptotic proteins in the biochemical pathways that will allow us to better understand how the cell makes the decision between life and death. No significant data are available related to occupational exposure of silica particles, which leads to the activation of caspases in silicosis. An equally important task is to study how these pathways are modified in occupational diseases such as silicosis in which apoptosis dysregulation contributes to the pathogenesis of silicosis. Moreover, caspase may be used as a key effector molecule in which inflammatory processes and proteases are involved in remodeling of the extracellular matrix. High-resolution technology is available for the measurement of its activity in the biological samples such as broncheoalveolar lavage fluid, cerebrospinal fluid and blood samples of silicotic individuals, which may provide a beneficial tool to better understand the cellular and molecular pathways in silicosis. In future, it can be used as an effector biomarker for the prognosis of silicosis. Fas and FasL pathways are also involved in silicosis, indicating dysregulation of autoimmunity after long-term exposure to silica by the dysregulation in Fas and FasL pathways, which alter the autoimmunity in silicosis. FasL could play a role in the modulation of silica-induced inflammation in humans, providing a clue for the pathogenesis and treatment of this common and life-threatening occupational disease. Studies are available related to oxidative stress, ROS, RNS, TNF and NF-kβ, but this area is less approached by the scientists so far in caspase activation in silicosis. Measurement of the activity of caspases, Fas/FasL and Apaf-1 may open a new area for the early diagnosis of silicosis. During the course of this pursuit, novel proteins that currently do not belong to the known family members of apoptosis may be identified as antiapoptotic proteins, such bcl-2, which may be used as an effective biomarker for the study of occupational diseases. This review may help in better understanding caspase activation in silicosis and establishment of suitable biomarkers for screening the occupational diseases. Finally, it is possible that these basic scientific discoveries on apoptosis may reveal logical strategies for the discovery of new tools for the early diagnosis and prognosis of silicosis.
